# Role of the *ABCE1* gene in human lung adenocarcinoma

**DOI:** 10.3892/or.2012.1646

**Published:** 2012-01-19

**Authors:** YI REN, YINGHUI LI, DALI TIAN

**Affiliations:** 1 Department of Thoracic Surgery, The Fourth Affiliated Hospital of China Medical University, Shenyan 110032; 2 Department of Thoracic Surgery, Liaoning Province Tumor Hospital, Shenyang 110042; 3 Department of Medical Genetics, College of Basic Medical Sciences, China Medical University, Shenyang 110001, P.R. China

**Keywords:** ATP-binding cassette transporter E1, lung adenocarcinoma, expression, siRNA

## Abstract

ATP-binding cassette transporter E1 (ABCE1), also known as RLI (RNase L inhibitor), is a new type of endoribonuclease inhibitor, which can specifically bind to RNase L and abolish its effect. ABCE1 binds to eIF2α and eIF5 to form a pre-translation initiation complex, suggesting its crucial role in cell growth, development and certain pathological processes. To probe the role of ABCE1 in the development and progress of human lung adenocarcinoma, we first detected the changes of its mRNA and protein expression in tissues, and found a high expression level of *ABCE1* in human lung adenocarcinoma tissues and metastatic lymph nodes, which was also correlated with clinical stages. Moreover, human lung adenocarcinoma A549 cells were infected with lentiviral vectors containing *ABCE1*-specific shRNA, and resulted in significant inhibition of cell growth. Using microarray assay, a number of differentially expressed genes were found after *ABCE1* suppression. Our results demonstrated the potential role of ABCE1 in human lung adenocarcinoma, which may provide some molecular basis for the mechanisms of development and progress of human lung adenocarcinoma, and help to find new pharmacological targets.

## Introduction

Lung cancer is the maligant tumor with the highest incidence world-wide, and adenocarcinoma is one of the major pathological types. Due to its unknown developmental mechanism, early metastasis and insensitivity to radiation and chemo-treatment, 5-year survival rate is very low. Therefore, to probe the newly found related genes is an important task to improve the prognosis of lung cancer.

ATP-binding cassette transporter E1 (*ABCE1*) gene is located at 4q31 and encodes a 68-kDa protein. The protein is composed of 599 amino acids, which is classified as an ABC transporter based on the sequence and organization of its ATP-binding domain, also known as nucleotide-binding folds (NBFs). The NBFs contain characteristic motifs, Walker A and B, signature C, Q-loop and H-loop. However, due to its lack of transmembrane (TM) domains, ABCE1 can not transport substrates across the membrane as other members of the family. ABCE1, also known as RLI (RNase L inhibitor), is a new type of endoribonuclease inhibitor, which can specifically bind to RNase L and abolish its effect (1). The 2′-5′ oligoadenylate (2–5A)/RNase L pathway is one of the enzymatic pathways induced by interferon and RNase L is the latent endoribonuclease which is activated by 2–5A and inhibited by RLI. This system has an important role in regulating viral infection. Additionally, variations in RNase L activity have been observed during cell growth and differentiation (2–4). Although ABCE1 may inhibit the interferon-induced 2–5A/RNase L, the expression of *ABCE1* mRNA is not influenced by interferon. Induction of RNase L by interferon through the 2–5A/RNase L pathway may result in RNA degradation and apoptosis (1,5). Moreover, ABCE1 protein binds to eIF2α and eIF5 to form a pre-translation initiation complex (6). Chen *et al* (5) have shown that inhibition of the *Xenopus* ABCE1 arrests growth at the gastrula stage of development, consistent with a block in translation. Suppression of ABCE1 expression by siRNA inhibits the proliferation of HEK 293 cells. These studies showed ABCE1 may be an important factor in cell growth, development and certain pathological processes.

In the present study, we aimed at assessing the role of ABCE1 in the development and progress of human lung adenocarcinoma. We first detected the expression of *ABCE1* mRNA and protein in lung adenocarcinoma tissues and metastatic lymph nodes. Subsequently, we constructed lentiviral vectors containing *ABCE1*-specific shRNA and infected human lung adenocarcinoma A549 cells to supress ABCE1 expression. Then cell proliferation was evaluated and differentially expressed genes were screened.

## Materials and methods

### Preparation of tissue specimens and cell culture

Lung and lymph node tissues (18 cases of lung adenocarcinoma tissues, 17 cases of paired normal lung tissues, and 15 cases of metastatic lymph nodes) were obtained from the Liaoning Province Tumor Hospital. All human tissue samples were collected with informed consent of the patients. In all the patients lung adenocarcinoma was confirmed by pathological diagnosis. Human lung adenocarcinoma A549 cells (originally obtained from American Type Culture Collection, ATCC) were maintained in RPMI-1640 medium (Sigma-Aldrich, St. Louis, MO, USA) at 37°C under 5% CO_2_, supplemented with 10% fetal bovine serum, 100 units/ml penicillin, and 100 μg/ml streptomycin.

### RT-PCR

Total RNA was isolated from different tissues or A549 cells using TRIzol reagent (Invitrogen, Carlsbad, CA, USA), and cDNA was synthesized with the cDNA Synthesis Kit (Roche, Basel, Switzerland). The primers for *ABCE1* and β-actin were: *ABCE1* 5′-TTGGTTGTGGGAAGTCGT-3′ (sense) and 5′-GCTTATGTAGTTAATGGGAGGT-3′ (antisense); β-actin, 5′-CTTCCTGGGCATGGAGTC-3′ (sense) and 5′-GCCGATCCACACGGAGTA-3′ (antisense). PCRs were carried out under optimized conditions. Agarose electrophoresis (2%) was used for detection. The integrated density values (IDV) were calculated with β-actin as an internal control.

### Western blot analysis

Cell lysis from tissues or A549 cells were extracted as usual, separated by SDS-polyacrylamide gel and transferred to polyvinylidene difluoride membranes (Millipore, Bedford, MA, USA). The membranes were then incubated with an anti-ABCE1 or β-actin monoclonal antibody (Sigma-Aldrich) diluted by 1:200, followed by incubation with a rabbit anti-mouse horseradish peroxidase conjugated IgG. The ECL Western blot analysis kit (Amersham, Italy) was used to observe the results.

### Vector production and infection

ABCE1 shRNA was synthesized and annealed using the following oligonucleotides: 5′-GATCCGCTACAGCGAGTACGTTTACCTGTGAAGCC ACAGATGGGGTAAACGTACTCGCTGTAGCTTTTTTG-3′ and 5′-AATTCAAAAAAGCTACAGCGAGTACGTTTACC CCATCTGTGGCTTCACAGGTAAACGTACCTGCTGTAG CG-3′. And the double-strand *ABCE1* shRNA was cloned into lentiviral pSC-GFP vectors encoding the green fluorescent protein (GFP). Then 293T cells were transfected using calcium phosphate with the VIRPAC packaging construct pCMV-dR8.74 and pMD2G. Supernatant containing lentiviral particles were collected every 72 h after transfection and collected by centrifugation. Titers (IU/ml) were the cells with fluorescent signals (percent)/5×1.5×10^5^×10^3^. Subsequently, lentiviral particles with MOI of 1×10^5^ IU/ml were directly added to 5×10^5^ A549 cells and cells were cultured for 72 h. Fluorescent intensity was detected under a microscope.

### MTT

Cell viability was examined by routine 3-(4,5-dimethylthiazol-2-yl)-2,5-diphenyltetrazolium bromide assay. A549 cells (1×10^6^) were placed in 96-well plates with RPMI-1640 in a final volume of 500 μl. The following day, cells were transfected with pSC-ABCE1. Then cell proliferation was assessed by MTT assay after 24, 48, 72, 96 or 120-h culture. Following incubation at 37°C for 3 h, the reaction was stopped by the addition of 150 μl DMSO. After the crystal dissolved, the absorbency of the samples was determined at 492 nm.

### Microarray

Total RNA was extracted with TRIzol reagent (Invitrogen). RNA quantity was measured by A260/A280 ratio. RNA quality was assessed using agarose electrophoresis. Samples with high-quality RNA were hybridized to human WG-6V3 Beadchip KIT expression microarray (Illumina, San Diego, CA) in accordance with the Illumina standard protocol, and the data were analyzed using Illumina BeadArray Reader.

### Statistical analysis

Experiments were performed a minimum of three times. Results represent the mean ± SD from three experiments. Representative results are depicted. We compared data using one-way ANOVA or t-test as appropriate, and defined statistical significance at p<0.05.

## Results

### Expression of ABCE1 mRNA and protein in human lung adenocarcinomas

**Figure 1:**
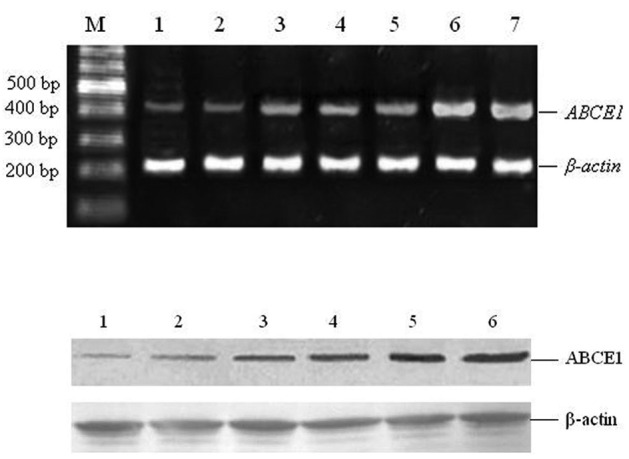
Expression of *ABCE1* in tissues. (A) *ABCE1* mRNA expression by RT-PCR. M, 100 bp DNA ladder; 1 and 2, normal lung tissues; 3–5, human lung adenocarcinoma tissues; 6 and 7, metastatic lymph nodes. (B) ABCE1 protein expression by Western blot analysis. 1 and 2, normal lung tissues; 3 and 4, human lung adenocarcinoma tissues; 5 and 6, metastatic lymph nodes.

To probe the role of *ABCE1* gene in human lung adenocarcinoma, we first detected its expression in human lung adenocarcinoma tissues. The results of RT-PCR and Western blot analysis showed that *ABCE1* mRNA and protein were expressed in lung adenocarcinoma tissues, normal lung tissues and metastatic lymph nodes (Fig. 1). By comparing the expression level in different tissues (17 cases of normal lung tissues, 18 cases of lung adenocarcinoma tissues and 15 cases of metastatic lymph nodes), we found that the expression of *ABCE1* mRNA and protein in cancer tissues were higher than in normal tissues (p<0.05) and lower than in metastatic lymph nodes (p<0.05) (Tables I and II).

Subsequently, we analyzed the association between the clinical stages (classified according to the 1997 TNM classification of UICC) and *ABCE1* expression of the 18 cases of lung adenocarcinoma. As shown in Tables III and IV, *ABCE1* mRNA and protein were differentially expressed in lung cancers of different clinical stages. The expression in cases of stage III was significantly higher than cases of stage I and II. And the expression in group of N1+2 was significantly higher than cases of N0.

### Construction and identification of lentiviral vectors containing ABCE1-specific shRNA and infection of human lung adenocarcinoma A549 cells

**Figure 2:**
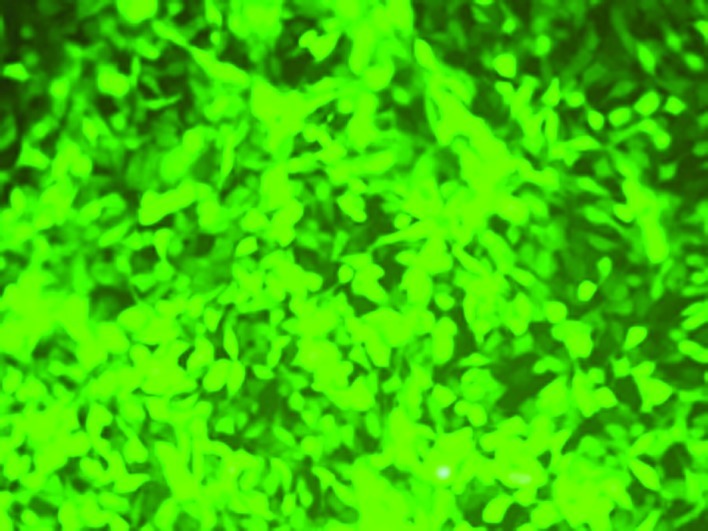
Detection of green fluorescence after transfection. pSC-ABCE1, pCMV-dR8.74 and pMD2G were co-transfected into 293T cells and green fluorescence was detected after 24 h.

Specific *ABCE1* shRNA fragment was cloned into the lentiviral pSC-GFP plasmid and confirmed by sequencing (termed pSC-ABCE1). Then pSC-ABCE1, pCMV-dR8.74 and pMD2G were co-transfected into incasing 293T cells. Twenty-four hours later, green fluorescence was detected under a fluorescent microscope (Fig. 2). Then the supernatant was collected to obtain lentivirus particles containing pSC-ABCE1. By infection of 293T cell, the virus titre was calculated as 1×10^5^ IU/ml.

**Figure 3:**
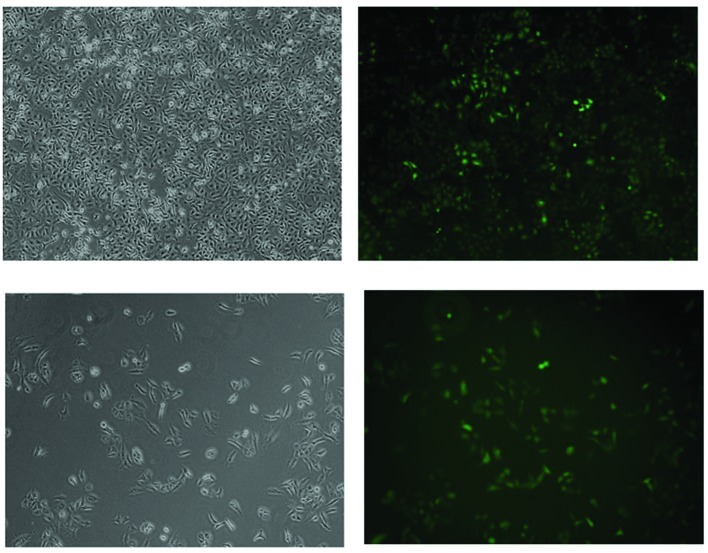
Detection of infection efficiency in A549 cells. By detecting the green fluorescence in A549 cells, the infection efficiency was over 90%.

Subsequently, the virus particles were used to infect human lung adenocarcinoma A549 cells. After 72-h infection, cells were detected under a fluorescent microscope and the infection efficiency was >90% by the signals of GFP (Fig. 3).

### Effect of ABCE1 inhibition on A549 cell proliferation

**Figure 4:**
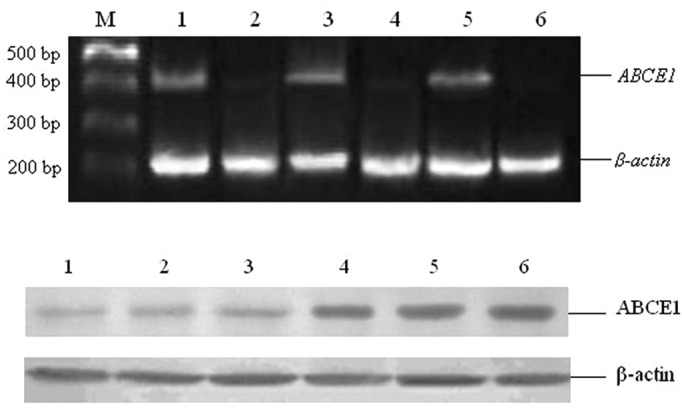
Inhibition of *ABCE1* expression by shRNA in A549 cells. RNA and protein were extracted from A549 cells infected with lentivirus expressing *ABCE1*-specific shRNA, and then RT-PCR and Western blot analysis were performed to detect *ABCE1* expression. (A) RT-PCR results: 1, 3 and 5, samples of control cells; 2, 4 and 6, samples of lentivirus-infected cells. (B) Western blot results: 1–3, samples of lentivirus-infected cells; 4–6, samples of control cells.

To assess the inhibition of *ABCE1* expression after infection, RNA and protein were extracted from control and lentivirus-infected cells. Then RT-PCR and Western blot analysis were carried out to detect the *ABCE1* mRNA and protein level. As expected, *ABCE1* expression was significantly repressed after infection (Fig. 4). RT-PCR showed the densitometry of ABCE1/β-actin was 0.286±0.07 in control cells, and that in infected cells was 0.023±0.003. The difference was statistically significant (p<0.05). The results of Western blot analysis also showed significant difference between the control and infected cells (the densitometry of ABCE1/β-actin was 0.499±0.097 and 0.109±0.004, respectively p<0.05).

**Figure 5:**
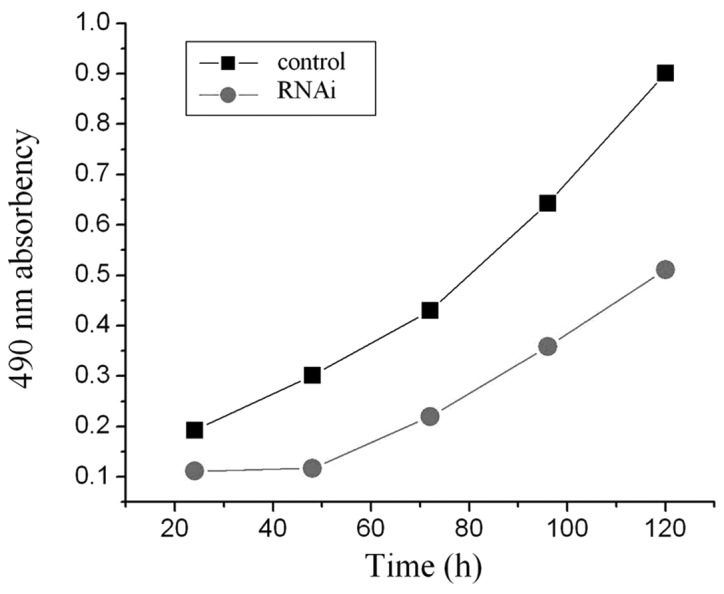
Growth curve of A549 cells.

Subsequently, MTT assay was carried out to assess the proliferation of A549 cells after *ABCE1* inhibition. Cells transfected with or without pSC-ABCE1 were analyzed after 24, 48, 72, 96 or 120-h culture. As shown in Fig. 5, the growth inhibition rate was 42.1, 61.2, 49.1, 44.3 and 43.2%, respectively, with the most obvious at 48 h, reaching 61.2% and the inhibition was significant (p<0.05).

### Differentially expressed genes after ABCE1 inhibition

RNA from control or lentivirus-infected A549 cells was prepared, and then expression chip was used to probe the effect of *ABCE1* inhibition. The results showed 476 evidently differentially expressed genes (149 genes were up-regulated and 327 ones were down-regulated), including 405 known protein-encoding genes, which could be divided into the following categories: inflammatory response factors, signal transduction proteins, metabolism-related proteins, cell proliferation, development, and differentiation-related factors, coagulation factors, gene transcription, translation and modification factors and cell adhesion and apoptosis-related molecules (Table V).

## Discussion

In the present study, we found a high expression level of *ABCE1* mRNA and protein in human lung adenocarcinoma tissues and metastatic lymph nodes, which was also correlated with clinical stages. The human lung adenocarcinoma A549 cells were infected with lentiviral vectors containing *ABCE1*-specific shRNA, and resulted in significant inhibition of cell growth. Subsequently, using microarray assay, a number of differentially expressed genes were found after *ABCE1* suppression. Our results demonstrated the potential role of ABCE1 in the development and progress of human lung adenocarcinoma.

It has been reported that mutations of RNase L gene may be correlated with human tumors. For example, RNase L mutation and reduction in catalytical activity are found in prostate cancer and allow tumor cells to escape a potent apoptotic pathway. Due to the effect of ABCE1 in inhibiting RNase L and its crucial role in regulating cell growth and proliferation, it is presumed to participate in the development and progress of human tumors. As ABCE1 is essential for translation initiation, and translation is a highly regulated process important to development and pathologies of cancer, tumor cells are thought to be more sensitive to the ABCE1 loss of function, making ABCE1 a potential target for therapeutics (7). In the present study, to probe the role of ABCE1 in human lung adenocarcinoma, we first detected its mRNA and protein expression in tissues. By comparing the expression level in different tissues (normal lung tissues, lung adenocarcinoma tissues and metastatic lymph nodes), we found that the expression of *ABCE1* mRNA and protein in cancer tissues were higher than in normal tissues, suggesting a possible role of ABCE1 in the development of lung adenocarcinoma. Our study showed, with the advancement of clinical stages of lung adenocarcinoma, *ABCE1* mRNA and protien expression were increased. The expression in tissues of stage III was significantly higher than that of stages I–II, and tissues in group N1+2 expressed at higher level of ABCE1 than that in group N0, which indicated high expression of ABCE1 may possibly induce the growth and metastasis of lung adenocarcinoma, and detection of ABCE1 expression may help to determine the progress of tumors.

Subsequently, to assess the function of ABCE1 in lung adenocarcinoma, we carried out siRNA assay. Lentiviral vectors containing *ABCE1*-specific shRNA were constructed and human lung adenocarcinoma A549 cells were infected. The ABCE1 expression was repressed, cell proliferation was greatly inhibited, with the most obvious effect at 48 h. Our result was in accordance with the report that down-regulation of ABCE1 inhibited proliferation of HEK 293 cells (7). These results demonstrated the role of ABCE1 in cell growth.

To probe the potential function of ABCE1 in lung adenocarcinoma, we performed microarray analysis to find the differentially expressed genes after ABCE1 silence. As expected, a number of genes were found to be up- or down-regulated, which encoding various types of proteins. The results showed the expression of 476 genes was evidently changed (149 genes were up-regulated and 327 ones were down-regulated), including 405 known protein-encoding genes, which could be divided into categories such as inflammatory response factors, signal transduction proteins, metabolism-related proteins, and cell proliferation-related factors, indicating the wide role of ABCE1 in the physiological and pathological processes. For example, the expression of GADD45 (growth arrest and DNA damage 45) was increased after ABCE1 silence. As a cell cycle-dependent protein, the expression of GADD45 is changed following the progress of cell cycle, at the highest in G1 phase and lower in S phase (8–11). It participates in maintaining genomic stability, DNA repair and inhibiting cell growth, which plays a crucial role in the block of cell transformation and maligant progress. Its abnormal expression is found in pancreatic cancer, and breast cancer (12,13). Our study showed silence of *ABCE1* gene induced GADD45 expression, and inhibited cell growth. Therefore, it is possible that ABCE1 repression blocked cells from entry to S phase, which may result in augmentation of GADD45. However, the accurate mechanism needs to be further probed.

Collectively, our results demonstrated abnormal expression of *ABCE1* gene in human lung adenocarcinoma and its effect in the proliferation of lung adenocarcinoma cells. A number of differentially expressed genes were found after *ABCE1* silencing. The present study may provide some molecular basis for the mechanisms of development and progress of human lung adenocarcinoma, and help to find new treatment targets.

## Figures and Tables

**Table I:** Relative expression of *ABCE1* mRNA expression in tissues.

Tissues	No. of cases	Relative *ABCE1* mRNA expression (densitometry of ABCE1/β-actin)	p-value
Normal lung tissues	17	0.113±0.003	
Lung adenocarcinoma tissues	18	0.473±0.05	<0.05
Metastatic lymph nodes	15	0.886±0.07	<0.05

**Table II:** Relative expression of *ABCE1* protein expression in tissues.

Tissues	No. of cases	Relative *ABCE1* protein expression (densitometry of ABCE1/β-actin)	p-value
Normal lung tissues	17	0.135±0.012	
Lung adenocarcinoma tissues	18	0.421±0.02	<0.05
Metastatic lymph nodes	15	0.84±0.036	<0.05

**Table III:** Relative expression of *ABCE1* mRNA expression in lung adenocarcinomas of different clinical stages.

	No. of cases	Relative *ABCE1* mRNA expression (densitometry of *ABCE1*/β-actin)	p-value
Stage			
I	5	0.175±0.021	
II	8	0.282±0.016	0.198
III	5	0.551±0.022	<0.05
Group			
N0	8	0.279±0.017	
N1+2	10	0.784±0.023	<0.05

**Table IV:** Relative expression of *ABCE1* protein expression in lung adenocarcinomas of different clinical stages.

	No. of cases	Relative *ABCE1* protein expression (densitometry of *ABCE1*/β-actin)	p-value
Stage			
I	5	0.193±0.023	
II	8	0.291±0.021	0.209
III	5	0.533±0.029	<0.05
Group			
N0	8	0.203±0.018	
N1+2	10	0.701±0.03	<0.05

**Table V:** Part of the differentially expressed genes after ABCE1 inhibition.

Genes	Description	Expression change
MTAP	Nucleotide and nucleic acid metabolism; S-methyl-5-thioadenosine phosphorylase activity	Up-regulated
GADD45	Activation of MAPKKK activity; apoptosis; cell differentiation	Up-regulated
Caspase-7	Apoptosis-related cysteine peptidase	Up-regulated
TANK	Signal transduction; metal ion binding	Up-regulated
ZNF14	Regulation of transcription, DNA-dependent; zinc ion binding	Up-regulated
P27KIP1	Regulation of cyclin dependent protein kinase activity, regulation of cell proliferation	Up-regulated
CIDEB	DNA damage response, signal transduction resulting in induction of apoptosis	Up-regulated
TIMP2	Metalloendopeptidase inhibitor activity	Down-regulated
TNFRSF1B	Cytokine and chemokine mediated signaling pathway, apoptosis	Down-regulated
CDH11	Homophilic cell adhesion; calcium ion binding	Down-regulated
LAMC1	Regulation of epithelial cell proliferation; cell adhesion	Down-regulated
TFF3	Defense response; digestion	Down-regulated
CD55	Complement activation, classical pathway; innate immune response	Down-regulated
CDK4	Cell proliferation; G1/S transition of mitotic cell cycle	Down-regulated
